# Supplementary figures and images for: Cell-cycle regulated transcription associates with DNA replication timing in yeast and human
Source: Genome Biol. 2013 Oct 7;14(10):R111. doi: 10.1186/gb-2013-14-10-r111 (PMC3983658; doi:10.1186/gb-2013-14-10-r111)

Figure S1

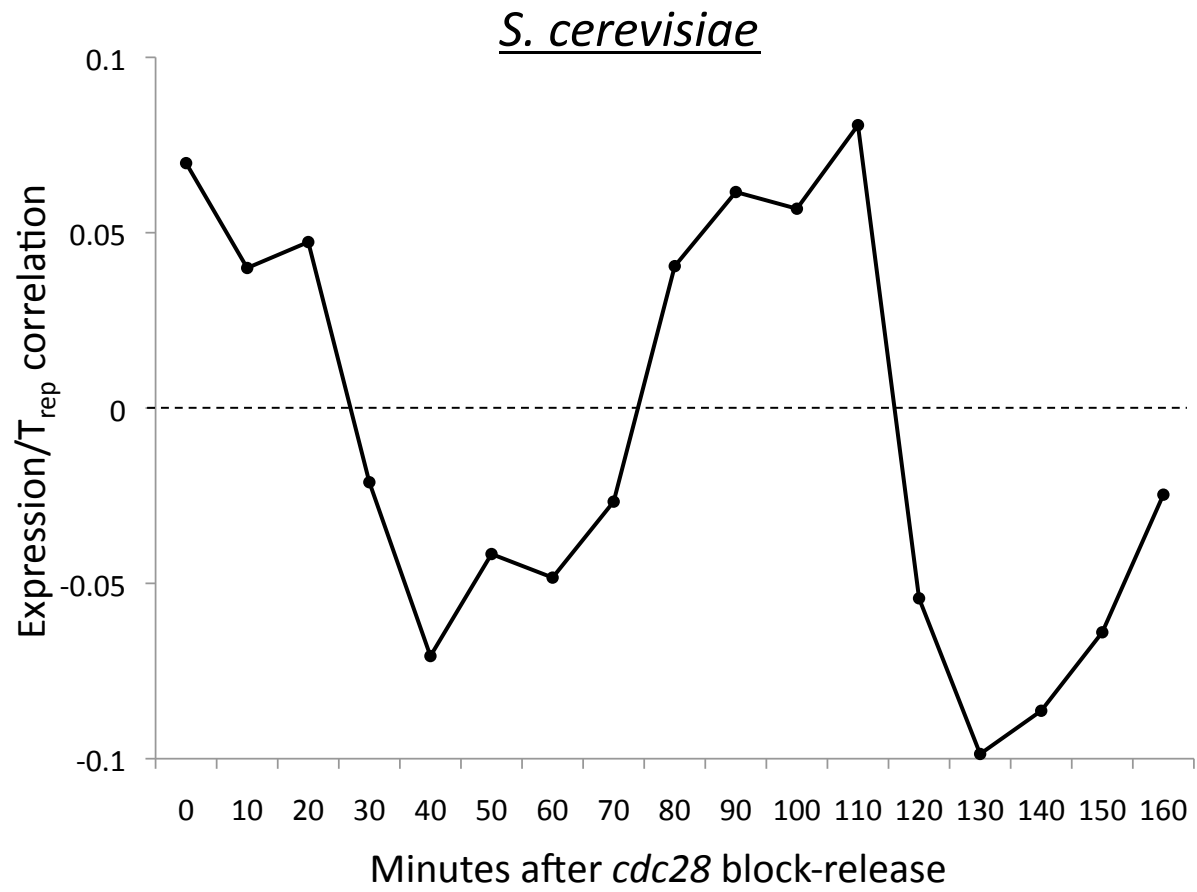

Figure S2

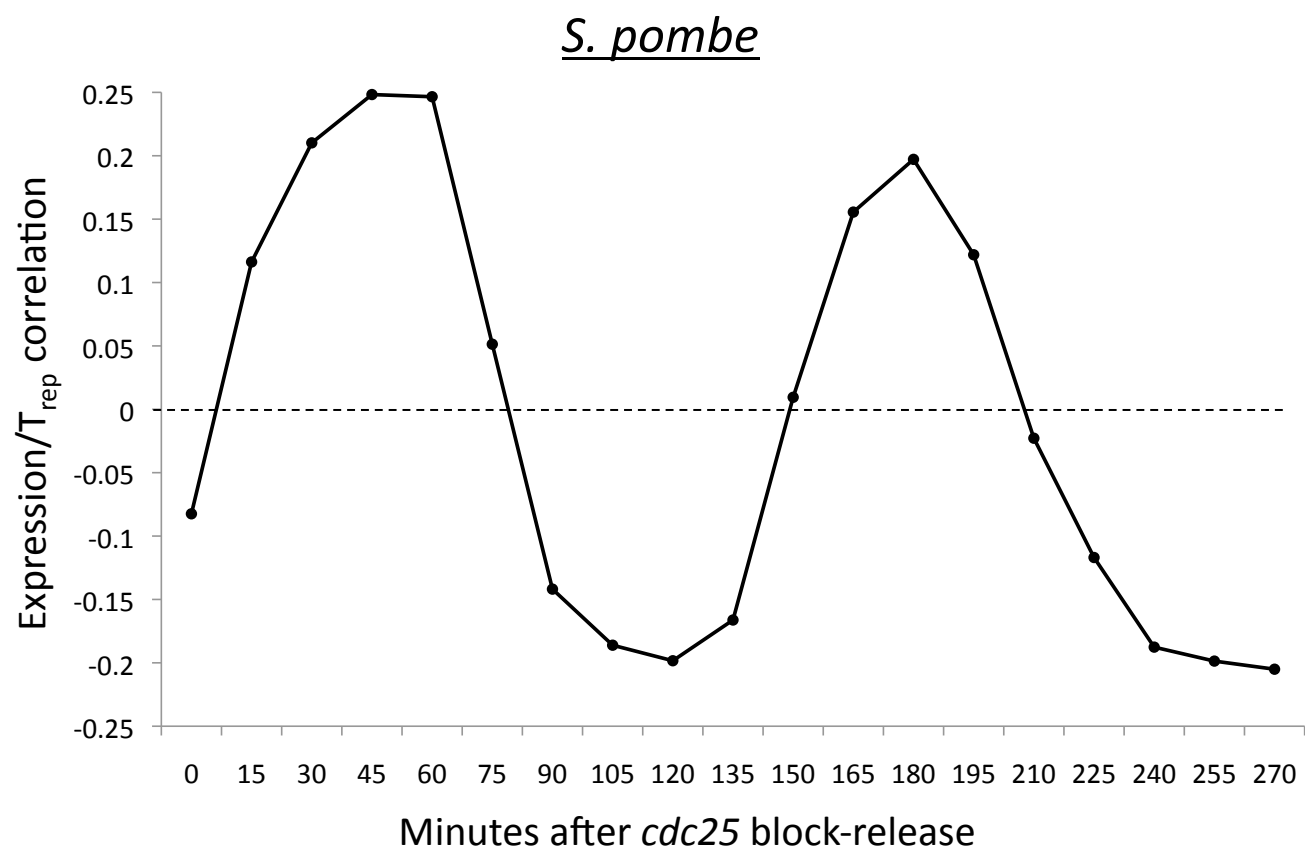

Figure S3

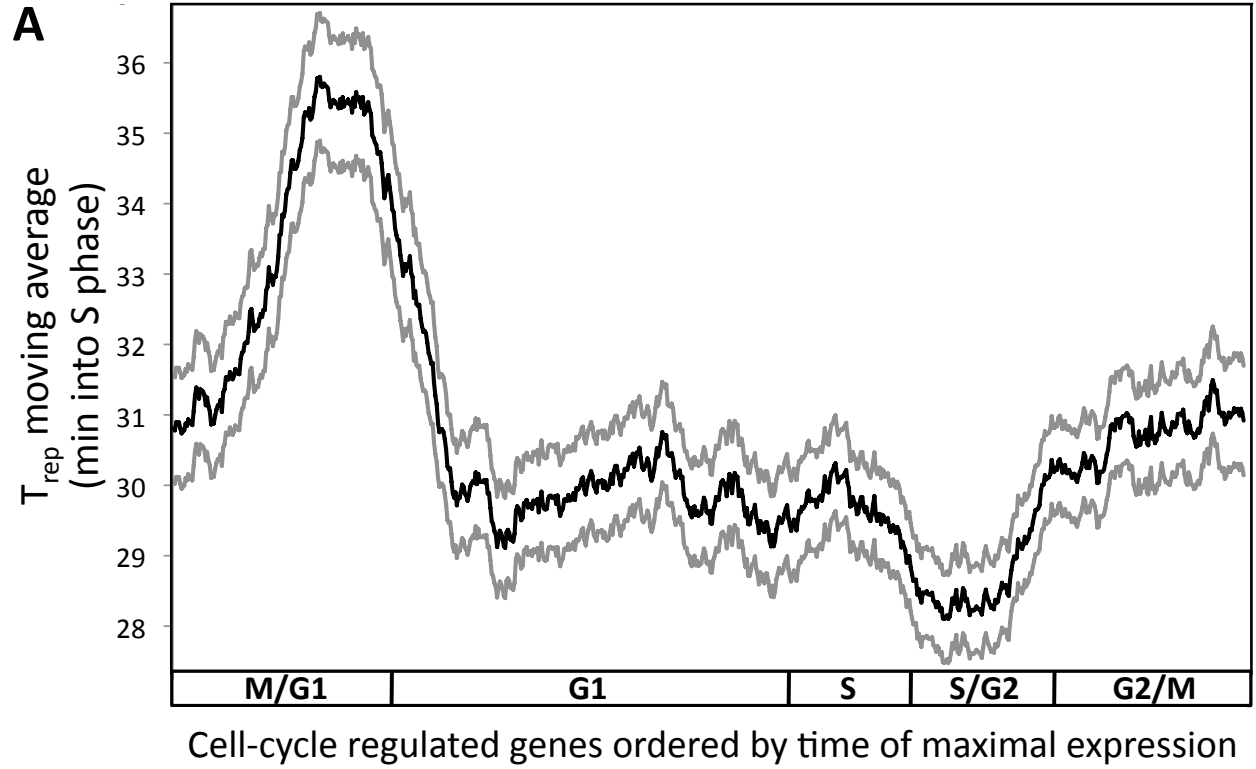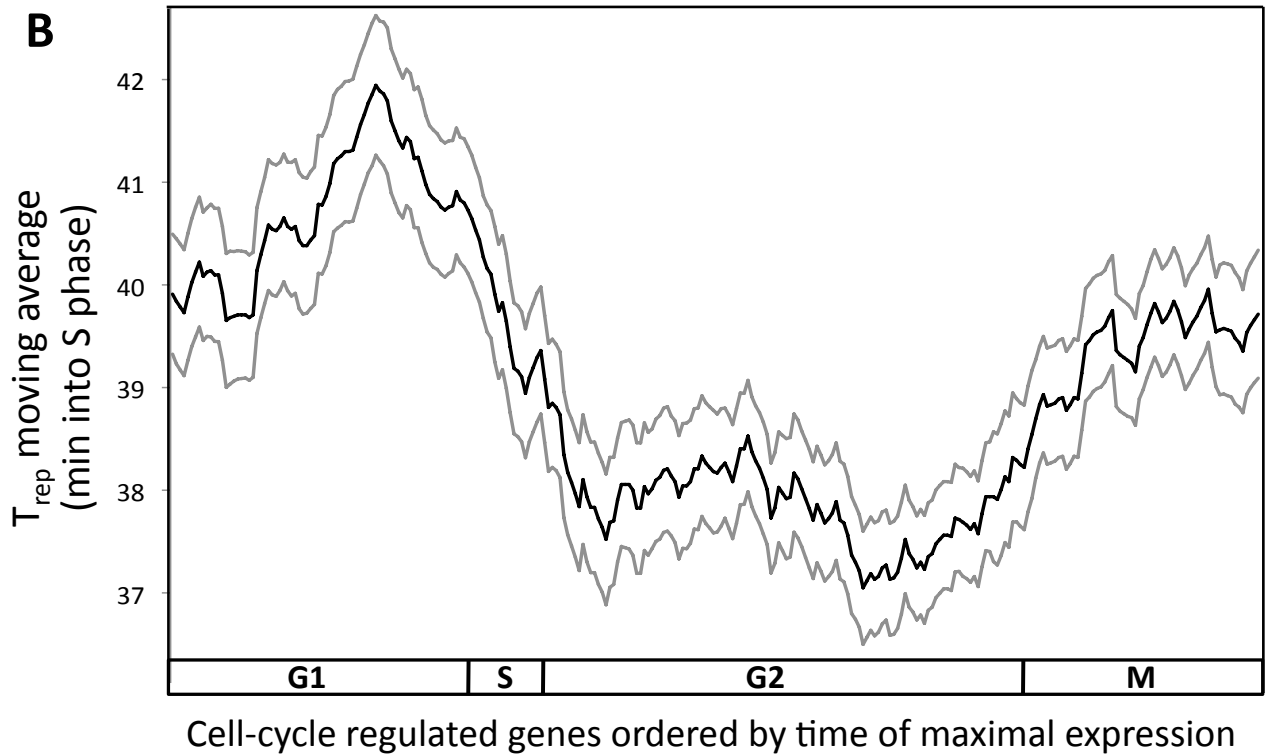

Figure S4

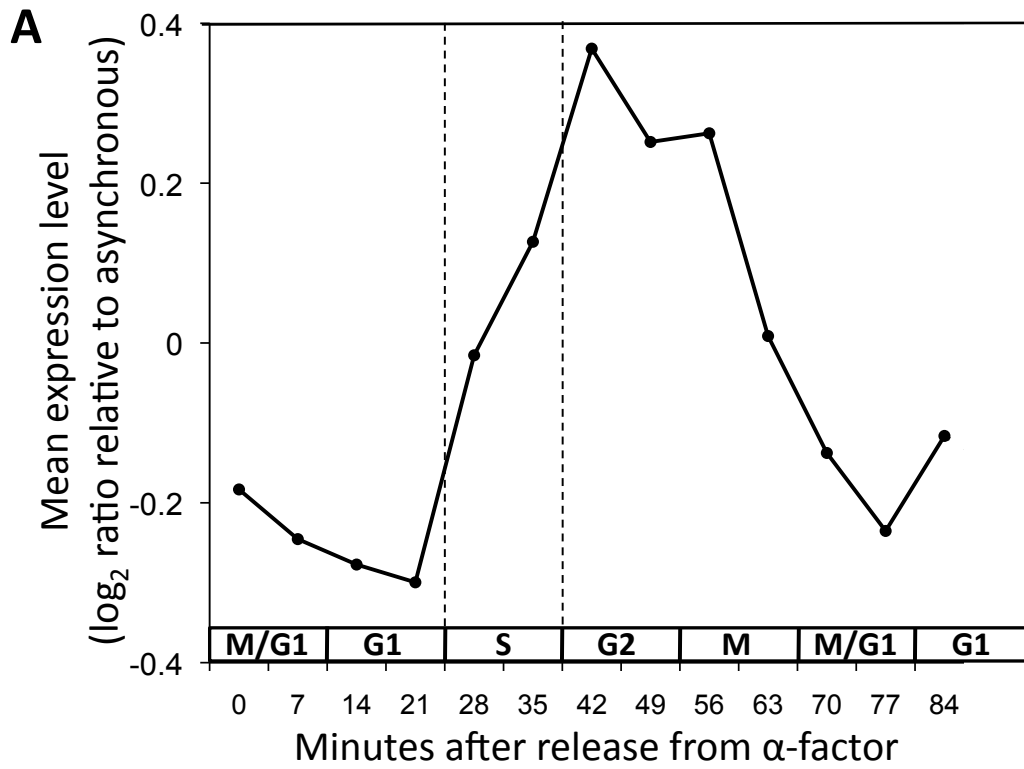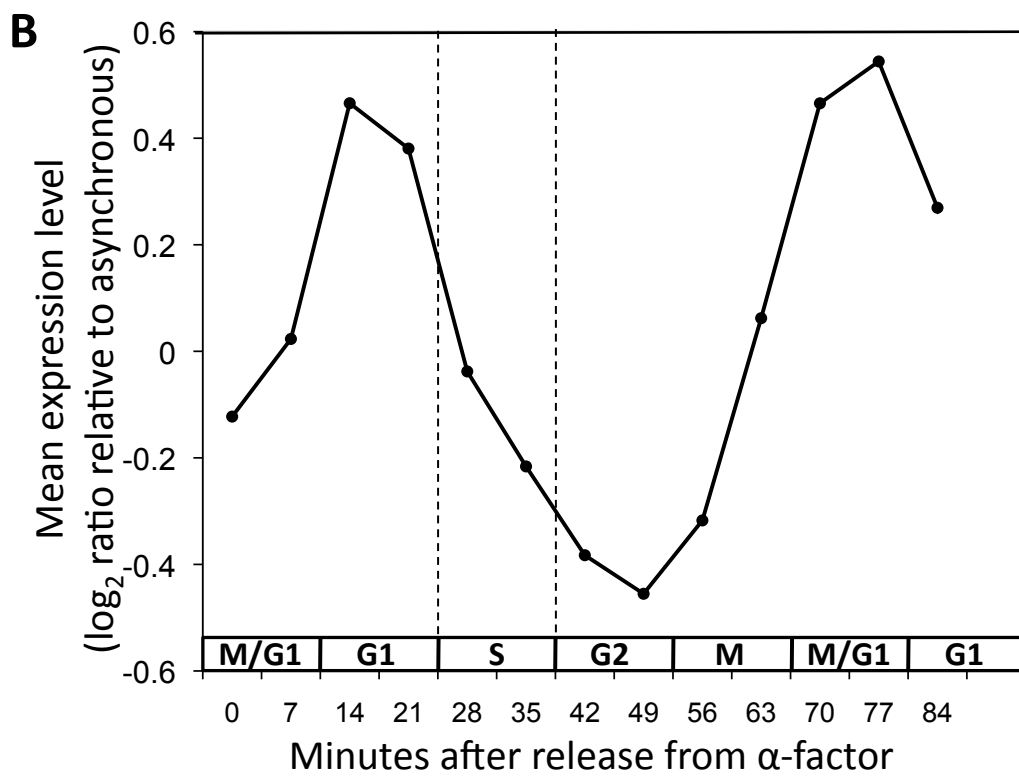

Figure S5

**A**

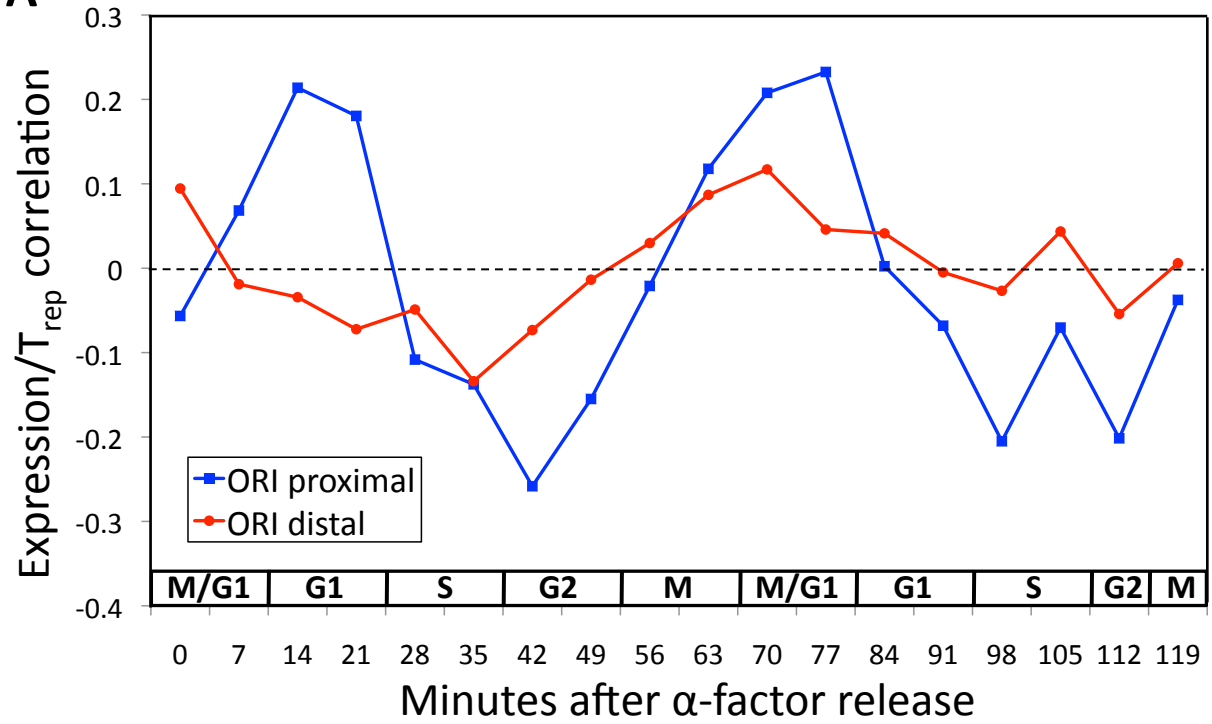

**B**

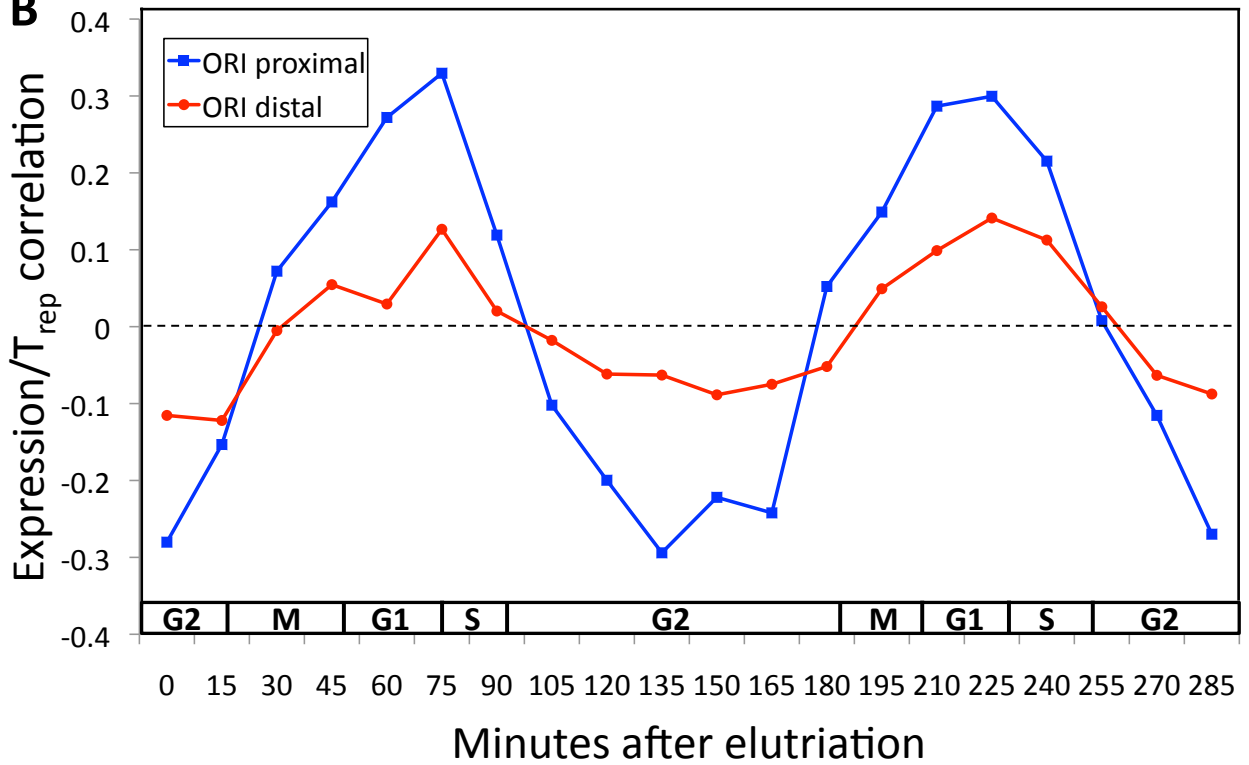

Supplement: Additional file 1: Figure S1 — Correlation analysis as in Figure 1B (left), but using a different method for synchronization of Sc (a temperature-sensitive cdc28 mutant). Figure S2. correlation analysis as in Figure 1B (right), but using a different method for synchronization of Sp (a temperature-sensitive cdc25 mutant). Figure S3. repeating the moving average analysis from Figure 1C, with standard errors shown for each point (grey lines) in (A)Sc and (B)Sp. Results suggest the differences between high and low windows are unlikely to be due to random fluctuations. Figure S4.(A) The mean expression level of 100 genes comprising the window with earliest Trep in Figure 1C (left) is plotted as a function of time in the cell cycle. The genes that reach a maximum mRNA level in G2 have their maximum rate of increase (and likely maximum rate of transcription) in S phase. (B) As for part (A), but showing the mean expression for the 100 genes in the window with the latest Trep in Figure 1C (left). The genes that reach a minimum mRNA level in G2 have their maximum rate of decrease (and likely minimum rate of transcription) in S phase. Figure S5. repeating the ORI proximal/distal analysis from Figure 3C using a cutoff of 7.5 kb to define ORI proximal in (A)Sc and (B)Sp. Results are qualitatively identical to Figure 3C. [file gb-2013-14-10-r111-S1.pdf]
